# Hydrogel Performance in Boosting Plant Resilience to Water Stress—A Review

**DOI:** 10.3390/gels11040276

**Published:** 2025-04-07

**Authors:** Gamareldawla H. D. Agbna, Syed Javaid Zaidi

**Affiliations:** 1 UNESCO-Chair in Desalination and Water Treatment, Center for Advanced Materials, Qatar University, Doha P.O. Box 2713, Qatar; g.agbna@qu.edu.qa; 2 Department of Agricultural Engineering, College of Agricultural Studies, Sudan University of Science and Technology, Shambat, Khartoum North P.O. Box 71, Sudan

**Keywords:** water retention, soil moisture management, drought stress mitigation, sustainable agriculture, crop resilience, polymeric materials, precision agriculture

## Abstract

Hydrogels have emerged as a transformative technology in agriculture, offering significant potential to enhance crop resilience, improve water use efficiency, and promote sustainable farming practices. These three-dimensional polymeric networks can absorb and retain water, making them particularly valuable in regions facing water scarcity and unpredictable rainfall patterns. This review examines the types, properties, and applications of hydrogels in agriculture, highlighting their role in improving soil moisture retention, enhancing nutrient delivery by, and increasing crop yield. The discussion extends to the economic and environmental implications of hydrogel use, including their potential to reduce irrigation costs by and minimize soil erosion. The review also explores the latest innovations in hydrogel technology, such as smart hydrogels and biodegradable alternatives, which offer new possibilities for precision agriculture and environmental sustainability. Despite promising benefits, challenges such as the higher cost of synthetic hydrogels, environmental impact, and performance variability across different soil types remain. Addressing these challenges requires a multidisciplinary approach that integrates advancements in material science, agronomy, and environmental policy. The future outlook for hydrogels in agriculture is optimistic, with ongoing research poised to refine their applications and expand their use across diverse agricultural systems. By leveraging the capabilities of hydrogels, agriculture can achieve increase in productivity, ensure food security, and move towards a more sustainable and resilient agricultural landscape.

## 1. Introduction

**Figure 1:**
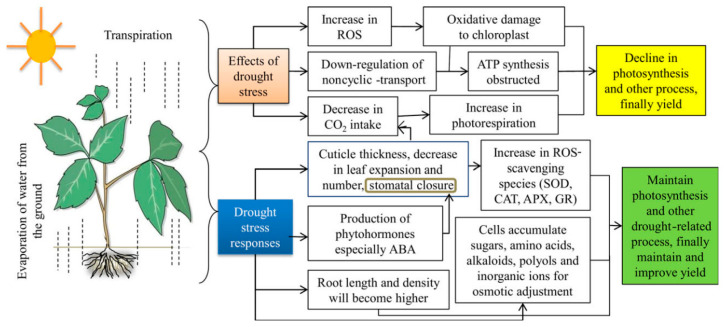
Negative impacts and adaptive responses of plants to drought stress [29,30].

Water stress, driven by factors such as climate change, population growth, and unsustainable agricultural practices, has become a critical challenge for global food security. Water stress occurs when the demand for water exceeds the available supply during a certain period, or when poor quality restricts its use, leading to adverse impacts on agricultural productivity [1]. Agricultural systems, especially those in arid and semi-arid regions, are increasingly vulnerable to water scarcity, resulting in reduced crop yields, soil degradation, and economic instability [1,2,3,4]. Figure 1 illustrates the negative impacts of drought stress on plants and their adaptive responses. Drought stress affects plants at various levels, leading to physiological changes such as stomatal closure, reduced photosynthesis, and oxidative stress, as well as biochemical and molecular adaptations including the activation of antioxidant defense mechanisms and stress-responsive gene expression. Plants employ various coping strategies, including osmotic adjustment, root system modification, and the synthesis of protective metabolites, to mitigate drought-induced damage. The abbreviations used in Figure 1 include ABA (Abscisic Acid), ROS (Reactive Oxygen Species), MAPK (Mitogen-Activated Protein Kinase), JA (Jasmonic Acid), SOD (Superoxide Dismutase), CAT (Catalase), APX (Ascorbate Peroxidase), and GR (Glutathione Reductase), which represent key physiological and molecular pathways involved in plant responses to drought stress.

In response to these challenges, innovative solutions are being explored to enhance water use efficiency and improve the resilience of crops to water-limited conditions [5]. One such solution is the use of hydrogels, which are highly absorbent polymers capable of retaining large volumes of water and releasing it gradually over time. Hydrogels have gained attention for their potential to improve soil moisture retention, reduce water loss through evaporation and deep percolation, and support plant growth during periods of drought [6,7].

Hydrogels are three-dimensional networks of hydrophilic polymers that can absorb several hundred times their weight in water [6]. Upon absorbing water, they swell to form a gel-like structure that retains water and releases it slowly as the surrounding soil dries out, thereby providing a continuous water supply to plants [8,9]. This property makes hydrogels particularly effective in sandy and coarse-textured soils, where water retention is typically low and water infiltration rates are high [10]. By enhancing soil moisture availability, hydrogels can reduce the frequency of irrigation, conserve water, and improve crop yields under water-stressed conditions [6].

The application of hydrogels in agriculture has been extensively studied, with numerous reports highlighting their effectiveness in improving plant resilience to drought [6,7,11]. For instance, research has shown that crops grown in hydrogel-amended soils exhibit better growth, higher biomass, and increased yields compared to those grown in untreated soils [12]. These benefits are particularly pronounced in regions with erratic rainfall patterns, where hydrogels help stabilize soil moisture levels and reduce the impact of dry spells [7,13]. Additionally, hydrogels have been found to improve soil structure by increasing porosity and reducing bulk density, which enhances root penetration and nutrient uptake [14,15].

Despite the clear advantages, the widespread adoption of hydrogels in agriculture faces several challenges. One major concern is the cost of hydrogel materials, which can be prohibitively expensive for small-scale farmers in developing countries [6]. Furthermore, the environmental impact of synthetic hydrogels, many of which are derived from non-biodegradable petrochemicals, raises concerns about long-term soil health and sustainability [16,17]. To address these issues, researchers are exploring the development of biodegradable hydrogels made from natural polymers such as cellulose, chitosan, and starch, which offer the potential to reduce environmental risks while maintaining the water-retention benefits of traditional hydrogels [18,19].

The role of hydrogels in modern agriculture extends beyond water retention. Recent studies have demonstrated that hydrogels can also serve as carriers for fertilizers, pesticides, and other agrochemicals, enabling the controlled release of these inputs in response to soil moisture levels [20,21,22,23]. This dual functionality not only enhances water use efficiency but also reduces nutrient leaching and minimizes environmental contamination [24,25]. Moreover, the integration of hydrogels with precision agriculture technologies, such as soil moisture sensors and automated irrigation systems, could further optimize water and nutrient management practices, leading to more sustainable and productive farming systems [13,26].

Given the increasing pressure on global water resources, the need for effective water management strategies in agriculture has never been more urgent [27]. Hydrogels represent a promising tool for enhancing plant resilience to water stress, particularly in regions prone to drought and water scarcity [6,15]. However, their successful integration into agricultural practices will require ongoing research and innovation to overcome the technical, economic, and environmental barriers that currently limit their use [6,28].

This review aims to provide a comprehensive analysis of the performance of hydrogels in boosting plant resilience to water stress. This review examines the types, properties, and applications of hydrogels in agriculture, highlighting their role in improving soil moisture retention, nutrient delivery, and crop yield and their mechanisms of action [7]. The discussion extends to the economic and environmental implications of hydrogel use, including their impact on water conservation, soil health, and the cost-effectiveness of farming operations [6,7]. In addition, the review will address the challenges associated with hydrogel use and identify potential future directions for research and development in this field. The review also explores the latest innovations in hydrogel technology, such as smart hydrogels and biodegradable alternatives, which offer new possibilities for precision agriculture and environmental sustainability [15,18].

To ensure a comprehensive and up-to-date review of hydrogel applications in agriculture, relevant articles were selected from scientific databases, including Web of Science, Google Scholar, Elsevier, SpringerLink, and Scopus. The selection focused on peer-reviewed journal articles, conference proceedings, and high-impact review papers published in the last decade, prioritizing studies that provide experimental insights into hydrogel performance in different agricultural settings.

## 2. Types and Properties of Hydrogels

Hydrogels have emerged as a transformative technology in agriculture, offering significant potential to enhance crop resilience, improve water use efficiency, and promote sustainable farming practices [7,31,32]. These three-dimensional polymeric networks possess exceptional water retention capabilities, making them particularly valuable in regions facing water scarcity and unpredictable rainfall patterns [33,34,35]. Hydrogels, which can absorb and retain up to 500 times their weight in water, are designed to release moisture gradually, providing plants with a consistent supply of water during periods of drought [18,36,37]. Hydrogels are a versatile class of materials known for their ability to absorb and retain large amounts of water, making them highly valuable in agricultural applications. The classification of hydrogels is multifaceted, involving their polymer origin, crosslinking mechanism, and interaction with water. Understanding these classifications is crucial for selecting the right hydrogel for specific agricultural needs, as different types of hydrogels offer varying benefits and limitations [16].

### 2.1. Hydrogel Structure and Classification

The structure of hydrogels is typically a three-dimensional network of hydrophilic polymers that can absorb and hold large quantities of water [18,38,39]. This structure enables hydrogels to swell in the presence of water and release it slowly over time, which is crucial for maintaining soil moisture [7,22,38]. The crosslinking within the polymer network determines the gel’s mechanical strength and its ability to retain water under various environmental conditions [31,33,40,41].

**Figure 2:**
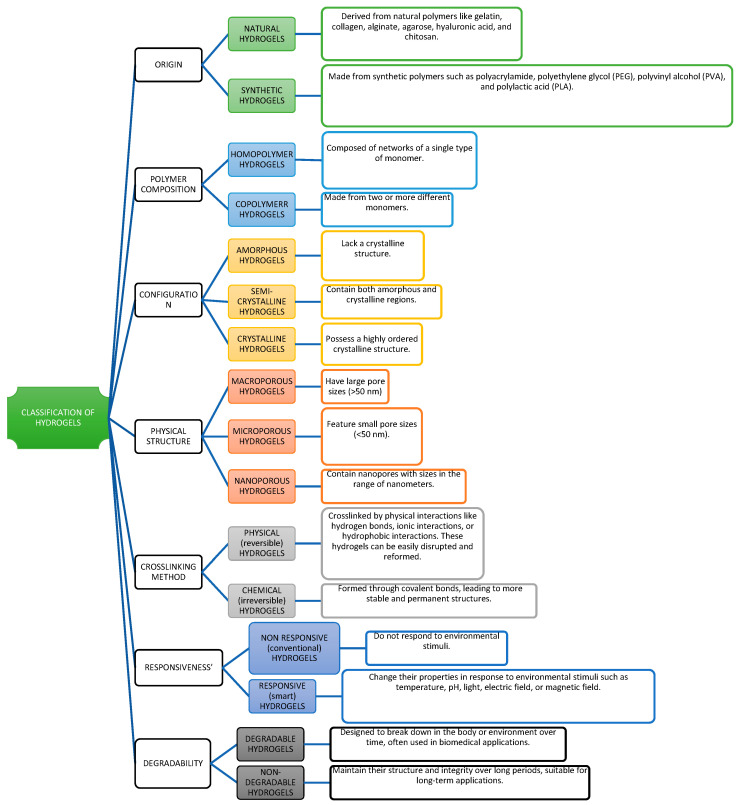
Classification of hydrogels [7,16].

Hydrogels can be classified based on their origin into natural, synthetic, and hybrid hydrogels (Figure 2). Natural hydrogels, derived from biopolymers like cellulose, chitosan, and starch, are biodegradable and environmentally friendly but may have lower mechanical strength [31,38,40,41]. Synthetic hydrogels, such as those made from polyacrylamide (PAM) and polyvinyl alcohol (PVA), offer greater durability and water retention but raise environmental concerns due to their persistence in soil [7,23,32,42]. Hybrid hydrogels combine the benefits of both natural and synthetic polymers, offering improved performance while maintaining some level of biodegradability [23,43].

#### 2.1.1. Classification Based on Polymer Origin

Hydrogels can be categorized based on their origin into natural and synthetic hydrogels (Table 1). This classification is important because the origin of the polymer determines not only the environmental impact but also the performance characteristics of the hydrogel in different applications [6].

1. Natural Hydrogels: Natural hydrogels are derived from biopolymers such as cellulose, starch, alginate, chitosan, and gelatin. These hydrogels are biodegradable, making them environmentally friendly and suitable for sustainable agricultural practices [19]. For instance, cellulose-based hydrogels have been effectively used in organic farming to improve soil moisture retention and enhance crop yields. Similarly, chitosan-based hydrogels, derived from the shells of crustaceans, are gaining popularity due to their biodegradability and antimicrobial properties, which can help protect plants from pathogens [18]. Despite their benefits, natural hydrogels generally have lower mechanical strength and water retention capacity compared to their synthetic counterparts, which can limit their use in certain harsh environments [24,36].
2. Synthetic Hydrogels: Synthetic hydrogels are made from synthetic polymers like polyacrylamide (PAM), polyvinyl alcohol (PVA), and polyethylene glycol (PEG). These materials are engineered to have high water absorption capacity, durability, and the ability to function under a wide range of environmental conditions [17]. Synthetic hydrogels are widely used in industrial agriculture due to their consistent performance and ability to support crop growth even in extreme conditions such as high salinity or drought. However, the non-biodegradable nature of most synthetic hydrogels has raised concerns about their long-term environmental impact, leading to ongoing research into developing biodegradable alternatives [16].

#### 2.1.2. Classification Based on Crosslinking Mechanism

Crosslinking is a critical process that determines the structural integrity, swelling behavior, and overall functionality of hydrogels. Hydrogels can be classified based on the type of crosslinking into physical (reversible) and chemical (irreversible) hydrogels, each offering unique properties and advantages depending on the intended application [51]. Table 2 provides a comparison of natural and synthetic hydrogels.

1. Physical (Reversible) Hydrogels: Physical hydrogels are formed through non-covalent interactions such as hydrogen bonding, ionic interactions, or hydrophobic forces. These hydrogels are characterized by their ability to undergo reversible phase transitions in response to environmental changes like temperature or pH [52,53]. For example, temperature-responsive hydrogels can transition between a sol (liquid) and gel (solid) state, which is particularly useful in controlled-release applications where the delivery of water or nutrients can be regulated by environmental conditions [9]. However, their mechanical strength is generally lower than that of chemically crosslinked hydrogels, limiting their use in applications that require long-term stability [19].
2. Chemical (Irreversible) Hydrogels: Chemical hydrogels are formed through covalent bonds between polymer chains, resulting in a stable and permanent network. These hydrogels are more robust and can maintain their structure and functionality even under extreme conditions such as high temperatures or salinity [6]. Chemical hydrogels are often used in long-term agricultural applications, such as soil conditioners that require durability and consistent performance over multiple growing seasons [42]. Due to their strong network, they are less responsive to environmental changes compared to physical hydrogels, but their ability to retain water and provide structural support makes them invaluable in challenging agricultural environments [10,54].

**Table 2:** Comparison of Natural and Synthetic Hydrogels.

Property	Natural Hydrogels	Synthetic Hydrogels	References
Biodegradability	High (inherent biodegradability)	Low (limited biodegradability)	[47,55]
Environmental Impact	Low (biocompatible, sustainable sources)	High (potential non-biodegradable waste)	[56]
Water Absorption Capacity	Moderate (varies by polymer type)	High (superior absorption due to synthetic design)	[57]
Durability	Moderate (weak mechanical strength)	High (stable, mechanically robust)	[47,55]

#### 2.1.3. Classification Based on Water Interaction

**Figure 3:**
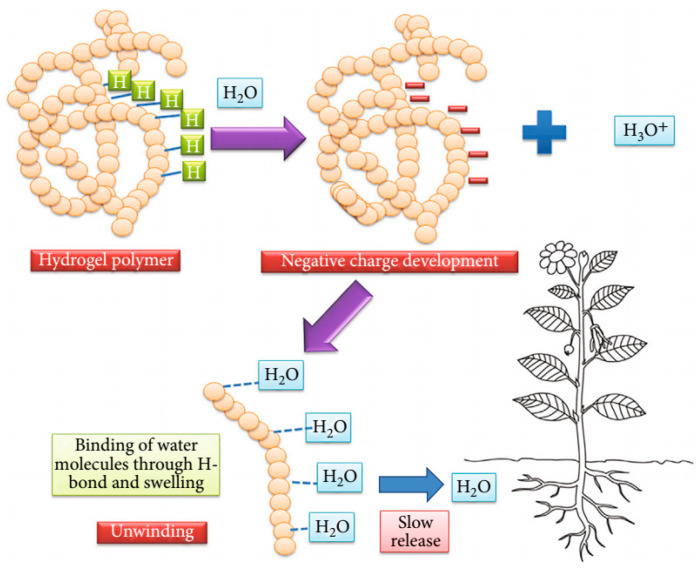
Structure of a hydrogel and Its water retention mechanism [6].

Hydrogels are also classified based on how they interact with water, which is essential for understanding their behavior in agricultural applications. The three primary classifications are free water, bound water, and intermediate water [58,59]. Figure 3, illustrates the three-dimensional network structure of a hydrogel, highlighting the absorption and retention of water within the polymer chains. Figure 3 also shows the gradual release of water as the surrounding soil dries out, demonstrating the hydrogel’s function as a water reservoir for plants.

1. Free Water (Non-Bound Water): Free water is loosely held within the hydrogel network and behaves similarly to bulk water. It is the first to be released when the hydrogel dries out and plays a critical role in the swelling and deswelling dynamics of the material [14].
2. Bound Water: Bound water is strongly associated with the polymer chains through hydrogen bonds or ionic interactions. This water remains within the hydrogel even under low humidity conditions, contributing to the stability and mechanical strength of the hydrogel [42].
3. Intermediate Water: Intermediate water exhibits properties between free and bound water. It is partially retained by the polymer network and influences the hydrogel’s overall performance, particularly in terms of water retention and release [18].

### 2.2. Properties of Hydrogels

**Figure 4:**
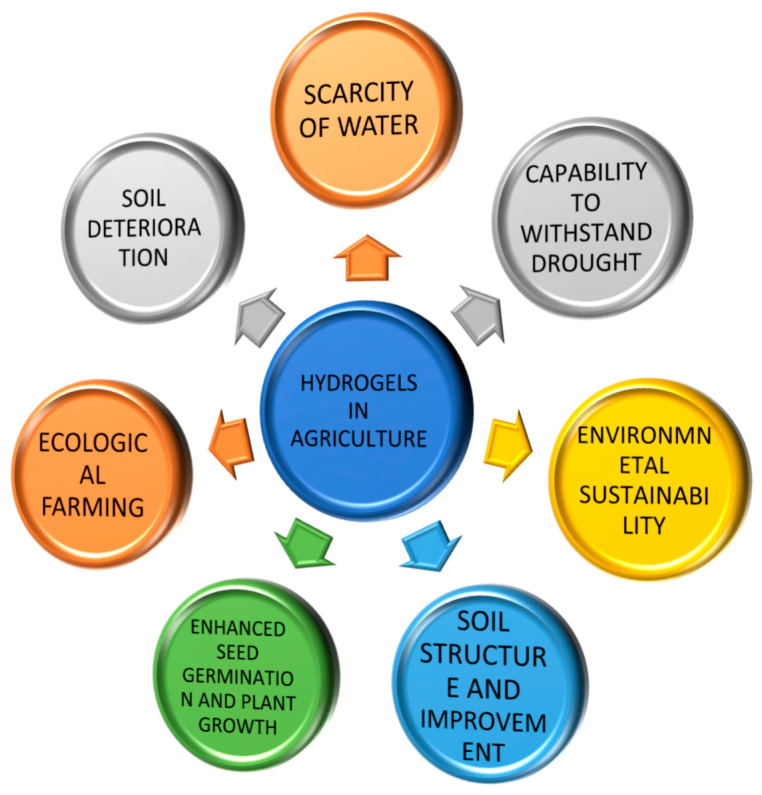
Advantages of hydrogels in agriculture.

The properties of hydrogels are determined by their chemical composition, crosslinking density, and the environmental conditions to which they are exposed. These properties include swelling capacity, mechanical strength, biodegradability, and environmental responsiveness [15]. Figure 4 depicts the various applications of hydrogels in agriculture, including their role in improving soil moisture retention, enhancing nutrient delivery, and supporting plant growth under drought conditions.

- Swelling Capacity: Hydrogels swell significantly when exposed to water, and their capacity to do so depends on the hydrophilicity of the polymer chains and the density of crosslinking. Higher crosslinking density generally leads to lower swelling capacity but greater mechanical strength [20,60]. This property is crucial in agriculture, as it determines how much water a hydrogel can store and subsequently release to the soil and plants [17].
- Mechanical Strength: The mechanical strength of hydrogels is influenced by their crosslinking density. Hydrogels with high crosslinking density exhibit greater mechanical stability, making them suitable for long-term applications where durability is essential [6]. In contrast, hydrogels with lower crosslinking density are more flexible and can absorb more water, but they may not be as durable under extreme conditions [16].
- Biodegradability: Biodegradability is a key property of natural hydrogels, which decompose over time without leaving harmful residues in the environment [18]. This property makes natural hydrogels particularly attractive for sustainable agricultural practices. In contrast, most synthetic hydrogels are not biodegradable, which raises concerns about their long-term environmental impact [61].
- Environmental Responsiveness: Hydrogels can be designed to respond to specific environmental triggers such as temperature, pH, or ionic strength. For example, temperature-responsive hydrogels undergo a phase transition at a specific temperature, allowing for controlled water release in response to changing conditions [62]. Similarly, pH-sensitive hydrogels can swell or contract based on soil pH, making them ideal for targeted delivery of agrochemicals [20,63].

## 3. Hydrogel Mechanisms for Water Retention

The primary value of hydrogels in agriculture lies in their exceptional ability to absorb and retain large quantities of water, releasing it gradually to support plant growth over time. This water retention capability is achieved through the unique three-dimensional network structure of the hydrophilic polymer chains that constitute hydrogels [16]. Understanding the mechanisms behind this water retention is necessary for optimizing the use of hydrogels in various agricultural applications. Moreover, hydrogels are known for their ability to retain large amounts of water relative to their size, a property that makes them incredibly useful in agricultural contexts [6,23,64,65]. Their water retention capacity helps in maintaining soil moisture levels, reducing the need for frequent irrigation, and providing plants with a steady supply of water during periods of drought [7,9,12,15,60]. In sandy soils, for example, hydrogels can increase water retention by up to 50%, significantly improving crop survival rates in arid regions [7,10,60,66]. Moreover, hydrogels can also reduce water runoff and soil erosion, further contributing to their effectiveness in sustainable agriculture [10,66,67,68,69].

### 3.1. Mechanism of Water Absorption and Release

Hydrogels absorb water through capillary action and osmotic pressure, swelling as they take in moisture [58,70,71,72,73]. The water release process is governed by several factors, including the polymer’s crosslinking density, external humidity, and the osmotic pressure between the hydrogel and the surrounding soil [7,32]. Hydrogels with low crosslinking density tend to release water more readily, as the polymer chains are more loosely connected, allowing water to diffuse out of the network with relative ease [21,33,48]. In contrast, hydrogels with higher crosslinking density retain water more tightly, providing a more sustained release over time.

Environmental conditions such as soil dryness play a crucial role in triggering water release, ensuring that plants receive moisture when needed most [16,61,74]. Temperature and soil pH also influence this process. Temperature-responsive hydrogels can undergo a phase transition when exposed to specific temperatures, causing them to either swell or shrink, thereby controlling water release [64]. Similarly, pH-sensitive hydrogels expand or contract in response to soil pH changes, making them ideal for precision agriculture applications where the timing and rate of water delivery are critical [75].

This controlled absorption and release mechanism makes hydrogels particularly useful in precision agriculture, where water availability can be tightly managed to optimize crop growth [7,26,29,74]. As summarized in Table 3, various factors influence the water retention and release capabilities of hydrogels, including polymer composition, crosslinking density, and environmental conditions. Understanding these interactions is essential for selecting the most suitable hydrogel formulations for specific agricultural environments, ensuring improved water availability, soil structure enhancement, and overall plant health.

### 3.2. Water Absorption and Swelling Behavior

Hydrogels are composed of hydrophilic polymer chains that attract and bind water molecules through hydrogen bonding, van der Waals forces, and other intermolecular interactions [6]. When hydrogels come into contact with water, they undergo a process known as swelling, in which the polymer chains expand to accommodate the absorbed water. The degree of swelling is influenced by several factors, including the polymer’s chemical composition, crosslinking density, and the ionic strength of the surrounding environment [20,63,73].

In agricultural soils, where moisture availability is often variable, the swelling capacity of hydrogels enables them to function as reservoirs, absorbing excess water during irrigation or rainfall and retaining it within their network [15]. As the soil dries out, the hydrogels gradually release the stored water, ensuring a consistent supply to plant roots. This slow-release mechanism is particularly beneficial in arid and semi-arid regions, where maintaining soil moisture is critical for crop survival [17].

### 3.3. Hydrogel Degradation and Longevity

**Figure 5:**
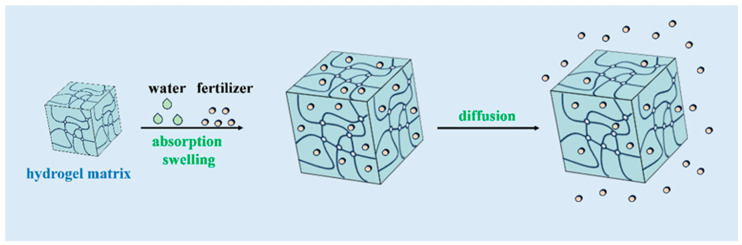
Hydrogel water and nutrient retention and release [80].

The effectiveness of hydrogels in agriculture also depends on their degradation rate and longevity in the soil. Synthetic hydrogels, such as those made from polyacrylamide, are generally more durable and can remain effective for several years [17]. However, concerns about their environmental impact have led to the development of biodegradable hydrogels derived from natural polymers like cellulose and starch [18]. These biodegradable hydrogels decompose over time, reducing the risk of soil contamination, but they may need to be replenished more frequently to maintain their water retention capabilities [16]. Figure 5 illustrates the cycle of water and nutrient absorption, retention, and release in hydrogels, from initial swelling upon contact with water to the gradual release of water into the soil as it dries out.

### 3.4. Applications of Water Retention Mechanisms in Agriculture

The water retention mechanisms of hydrogels have numerous applications in agriculture. For instance, hydrogels can be used in drought-prone areas to extend the time between irrigation events, conserving water and reducing the labor required for watering crops [6,7]. Additionally, hydrogels can be integrated into seed coatings to improve germination rates by ensuring that seeds have access to moisture even in dry soils [81,82,83]. The controlled release of water from hydrogels also helps to prevent waterlogging, which can occur when too much water is applied to crops too quickly [6].

In precision agriculture, hydrogels can be used in combination with soil moisture sensors and automated irrigation systems to optimize water use. By monitoring soil moisture levels in real-time, these systems can adjust irrigation schedules based on the moisture content provided by the hydrogels, ensuring that crops receive the right amount of water at the right time [19]. This integration of hydrogels with advanced farming technologies represents a significant step toward more sustainable and efficient agricultural practices.

## 4. Applications of Hydrogels in Agriculture

Hydrogels have found a wide range of applications in agriculture due to their ability to absorb, retain, and slowly release water, making them invaluable in water-scarce environments. Their use extends beyond mere water retention, encompassing soil conditioning, nutrient delivery, seed coating, and erosion control. This section explores the various ways hydrogels are applied in agriculture, highlighting their benefits, challenges, and potential for improving crop productivity and sustainability.

### 4.1. Soil Moisture Enhancement

One of the primary applications of hydrogels in agriculture is the enhancement of soil moisture retention. In regions prone to drought or with irregular rainfall patterns, maintaining adequate soil moisture is critical for plant growth. Hydrogels can absorb water during irrigation or rainfall and gradually release it into the soil as it dries out, ensuring a steady supply of moisture to plant roots [16]. This slow-release mechanism reduces the frequency of irrigation, conserves water, and mitigates the effects of drought, leading to more stable crop yields even under challenging conditions [17].

The effectiveness of hydrogels in enhancing soil moisture has been demonstrated across various soil types. In sandy soils, where water infiltration is rapid and retention is low, hydrogels significantly improve water-holding capacity, reducing water loss through deep percolation [6,8]. In clayey soils, hydrogels help prevent waterlogging by absorbing excess water and releasing it as needed, thereby maintaining optimal soil moisture levels [6]. These properties make hydrogels versatile tools for improving soil structure and function across diverse agricultural settings. As shown in Table 4, the impact of hydrogels on soil moisture retention varies across different soil types, influencing their effectiveness in improving water availability and plant growth.

### 4.2. Nutrient Delivery and Fertilizer Efficiency

In addition to their water retention capabilities, hydrogels are increasingly used as carriers for fertilizers and nutrients. By encapsulating nutrients within their polymer network, hydrogels can create controlled-release systems that deliver nutrients in response to soil moisture levels [7,22,82]. This approach not only improves fertilizer use efficiency but also reduces nutrient leaching, which is a common problem in conventional fertilization practices [42]. Nutrient-loaded hydrogels can be tailored to release specific nutrients at rates that match the growth stages of crops, ensuring that plants receive the right amount of nutrients when they need them most [40].

**Figure 6:**
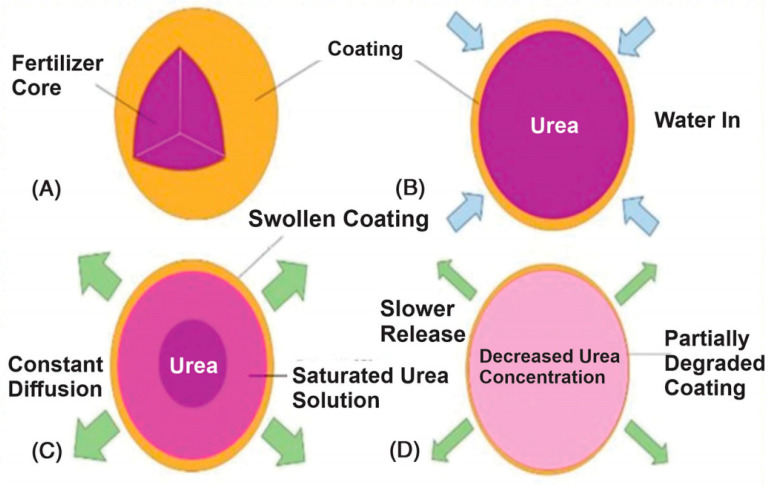
Controlled nutrient release from hydrogel-encapsulated fertilizers [65]. (**A**) Granule of a controlled-release fertilizer. (**B**) Initial phase involving water infiltration through the coating into the core. (**C**) Accumulation of internal pressure leads to a steady release of nutrients into the surroundings. (**D**) Final stage where the concentration gradient diminishes, causing a reduced release rate.

Studies have shown that hydrogels can significantly enhance the availability of essential nutrients such as nitrogen, phosphorus, and potassium, leading to better crop growth and higher yields [88]. For example, the integration of hydrogels with urea-based fertilizers has been shown to increase nitrogen use efficiency by reducing volatilization and leaching losses [19,89,90]. Similarly, hydrogels loaded with micronutrients like zinc and iron have been used to address specific nutrient deficiencies in crops, improving overall plant health and productivity [91]. Figure 6 shows the mechanism of how hydrogels release encapsulated nutrients into the soil in response to moisture conditions. It shows how the nutrients are gradually made available to plant roots, reducing the need for frequent fertilization and minimizing nutrient runoff.

### 4.3. Seed Coating and Germination Enhancement

Hydrogels are also used in seed coating to improve germination rates and seedling establishment. By coating seeds with hydrogels, farmers can ensure that seeds have immediate access to moisture, even in dry soils [6]. The hydrogel coating absorbs water from the surrounding soil and keeps it in close proximity to the seed, providing a favorable microenvironment for germination [15]. This is particularly beneficial in arid and semi-arid regions, where water availability is a major constraint to successful crop establishment.

Seed coating with hydrogels has been shown to improve the germination rate of a wide range of crops, including cereals, legumes, and vegetables [82,83]. The hydrogel coating not only provides moisture but can also be infused with nutrients, growth stimulants, or protective agents, further enhancing seedling vigor and reducing the risk of disease [87]. This approach is especially useful for direct seeding methods, where seeds are sown directly into the field without prior germination in nurseries [83]. Table 5 shows the benefits of hydrogel seed coating across different crops.

### 4.4. Erosion Control and Soil Stabilization

In addition to their role in water retention and nutrient delivery, hydrogels are also used in erosion control and soil stabilization. Hydrogels help bind soil particles together, reducing the risk of erosion caused by wind and water [16]. This is particularly important in areas with loose, sandy soils or steep slopes, where erosion can lead to significant soil loss and degradation [17]. By improving soil cohesion, hydrogels contribute to maintaining soil structure and fertility, supporting sustainable land management practices.

Hydrogels are often applied in combination with other soil stabilizers, such as mulch or geotextiles, to enhance their effectiveness. In agricultural settings, hydrogel-treated soils have shown improved resistance to erosion, particularly during heavy rainfall events [69]. This application is also beneficial for reforestation and land reclamation projects, where soil stabilization is critical for the successful establishment of vegetation [7].

### 4.5. Integration with Precision Agriculture

The integration of hydrogels with precision agriculture technologies represents a significant advancement in water and nutrient management. Precision agriculture involves the use of data-driven tools and technologies to optimize farming practices, and hydrogels fit well into this approach by providing targeted moisture and nutrient delivery [7,26]. For instance, hydrogels can be used in conjunction with soil moisture sensors to fine-tune irrigation schedules, ensuring that crops receive the exact amount of water they need based on real-time data [19].

In addition, hydrogels can be integrated into automated irrigation systems, allowing for the precise delivery of water and nutrients to specific areas of a field [9]. This reduces water waste and ensures that resources are used efficiently, which is particularly important in regions with limited water availability. The use of hydrogels in precision agriculture not only enhances crop performance but also contributes to more sustainable farming practices by minimizing inputs and reducing environmental impact [42].

## 5. Impact of Hydrogels on Plant Growth and Resilience

Hydrogels have a profound impact on plant growth and resilience, particularly in environments where water availability is limited. By improving soil moisture retention, nutrient availability, and overall soil structure, hydrogels contribute to enhanced plant growth, better root development, and increased crop yields. This section explores the various ways in which hydrogels influence plant health, focusing on their role in supporting resilience to environmental stressors such as drought and nutrient deficiency.

### 5.1. Improved Root Development and Biomass Production

One of the most significant impacts of hydrogels on plant growth is their ability to enhance root development. Hydrogels create a more favorable soil environment by maintaining consistent moisture levels, which encourages deeper root penetration and more extensive root systems [6]. Studies have shown that plants grown in hydrogel-amended soils exhibit greater root biomass compared to those grown in untreated soils. This improved root structure allows plants to access water and nutrients more effectively, particularly during dry periods [17].

For example, research on maize has demonstrated that the application of hydrogels increases root length and density, leading to better water uptake and higher biomass production [15]. Similarly, in wheat, hydrogel use has been associated with enhanced root growth, which contributes to improved drought tolerance and higher yields [7]. Table 6 illustrates the impact of hydrogels on root development across different crops.

### 5.2. Enhanced Plant Resilience to Drought

Hydrogels play a critical role in enhancing plant resilience to drought by maintaining soil moisture levels during dry spells. The water retention capability of hydrogels ensures that plants have access to moisture even when external water sources are scarce [17]. This is particularly important in arid and semi-arid regions, where drought conditions can severely limit crop production.

**Figure 7:**
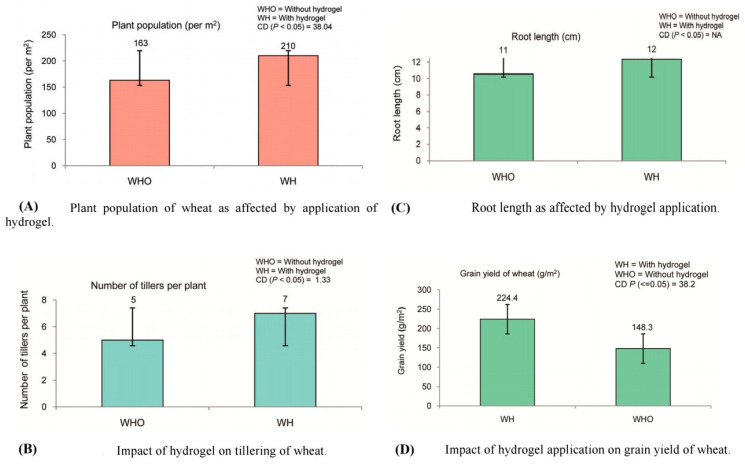
Comparison of plant growth with and without hydrogel application under drought conditions. (**A**) Plant population of wheat as affected by application of hydrogel. (**B**) Impact of hydrogel on tillering of wheat. (**C**) Root length as affected by hydrogel application. (**D**) Impact of hydrogel application on grain yield of wheat [74,104].

Numerous studies have highlighted the effectiveness of hydrogels in improving drought tolerance. For instance, in a study on peanut crops, the use of hydrogels resulted in a 30% increase in yield under drought conditions compared to untreated crops [103]. Similarly, in rice, hydrogels have been shown to reduce the negative effects of water stress, leading to higher grain yield and better overall plant health [6]. Figure 7 represents a comparison between plants grown with hydrogel treatment and those without under drought conditions. It highlights the differences in growth, plant population, tillering, root length, and overall grain yield, emphasizing the positive impact of hydrogels on drought resilience. This study was conducted in the Raipur Block of Dehradun district, Uttarakhand, India, at an elevation of 680 m above sea level. The experimental soil was classified as sandy loam with 46.4% sand, 37.6% silt, and 16% clay. The region experiences an average annual rainfall of approximately 1600 mm, with a mean maximum annual temperature of 30.2 °C and a minimum of 10 °C. The wheat variety used in the study was HS-507, selected for its adaptability to the North West Himalayan region. Hydrogel was applied at a rate of 5 kg ha⁻^1^ using the broadcasting technique. The study compared wheat growth under two treatments: with hydrogel (WH) and without hydrogel (WHO), with each treatment replicated ten times in field conditions. The results demonstrated that hydrogel application significantly enhanced plant population, effective tillering, and overall crop yield by improving soil moisture retention and mitigating drought stress [104].

### 5.3. Influence on Crop Yields and Quality

Hydrogels significantly enhance crop yields and quality by addressing critical limitations in plant growth, particularly under water-stressed conditions. Their ability to retain soil moisture and optimize nutrient delivery creates a stable environment for plants to thrive, directly translating to improved productivity. For instance, in mandarin cultivation, hydrogel application at 750 g/tree combined with 75% irrigation increased yield and reduced sun-burned fruits, with the highest yield-increasing percentage observed in this treatment [105]. Similarly, chickpea seed treatment with SPG1118 hydrogel at 2.5 kg/ha resulted in a 17.57 q/ha increase in seed yield and 97.33% germination rates, outperforming untreated controls by mitigating moisture loss and ensuring consistent root hydration [106].

Beyond yield, hydrogels elevate produce quality by stabilizing growth conditions. In tomato cultivation, hydrogel-treated seeds produced larger fruit size and higher lycopene content, critical for both nutritional value and marketability [105]. For strawberries, hydrogel coatings improved fruit firmness and sugar content, leading to a 20% higher marketable yield under drought stress [105]. These quality improvements stem from hydrogels’ capacity to maintain consistent soil moisture, preventing premature senescence and supporting optimal metabolic activity.

The mechanisms driving these benefits include moisture regulation, where hydrogels act as reservoirs to release water gradually during dry periods, and nutrient delivery, as encapsulated hydrogels can supply micronutrients (e.g., Zn, Mn) and macronutrients (e.g., N, P, K) to enhance plant vigor. For example, hydrogels in sandy soils can increase water retention by up to 40%, addressing issues related to water scarcity and soil water availability [10,60]. Additionally, hydrogels create a physical barrier against soil-borne pathogens like *Fusarium*, reducing disease incidence and preserving yield quality [107].

In yellow passion fruit cultivation, combining hydrogel with mulch (e.g., 10 g/plant + plastic mulch) significantly improved soil moisture retention and vine growth, leading to higher yields and net benefits compared to untreated controls [108]. This demonstrates hydrogels’ role in optimizing resource utilization under water-stressed conditions. Furthermore, studies highlight hydrogels’ ability to regulate soil water availability, improve nutrient absorption efficiency, and enhance yield under drought conditions [6,107].

### 5.4. Long-Term Benefits and Sustainability

The long-term use of hydrogels in agriculture offers sustainable benefits beyond immediate yield improvements. Hydrogels contribute to soil health by enhancing soil structure, reducing erosion, and preventing nutrient leaching [69]. Over time, the consistent use of hydrogels can lead to improved soil fertility, better water management, and more sustainable farming practices.

Moreover, the environmental benefits of using biodegradable hydrogels are significant. Unlike traditional chemical amendments, biodegradable hydrogels break down naturally in the soil, leaving no harmful residues behind [16]. This makes them an environmentally friendly option for farmers seeking to reduce their ecological footprint while maintaining high levels of productivity.

### 5.5. Impact of Synthetic Hydrogels on Soil Microbial Communities

Synthetic hydrogels significantly influence soil microbial communities through physical, chemical, and temporal mechanisms, reshaping microbial activity and nutrient cycling.

1. Physical Confinement and Metabolic Shifts

Encapsulating microbes in synthetic hydrogels like polyethyleneglycol-dimethacrylate (PEGDMA) alters their metabolic pathways and spatial interactions. For instance, Ruminiclostridium cellulolyticum co-encapsulated with cellulose in PEGDMA exhibited delayed cellulose degradation (13-day lag) and shifted fermentation products from ethanol-lactate mixtures to acetate dominance [109]. This confinement mimics soil aggregates, restricting microbial mobility and creating localized chemical gradients (e.g., oxygen levels), which modulate redox conditions and resource accessibility [7,109]. Transparent hydrogels enable real-time visualization of microbial colonization on substrates, bridging microscale behaviors to ecosystem-scale carbon cycling [109].

2. Water and Nutrient Dynamics

Hydrogels enhance soil water retention, amplifying microbial respiration in moisture-limited environments. In Mediterranean soils amended with TerraCottem hydropolymers, microbial respiration spiked initially but equilibrated over time, with no significant difference between low (1.5 kg/m^3^) and high (3.0 kg/m^3^) doses [110]. However, high hydrogel doses increased nitrogen leaching, suggesting reduced microbial nitrogen immobilization efficiency under prolonged hydration [110]. Synthetic hydrogels also suppress nitrification and denitrification by restricting ammonium mobility, preserving nitrogen in agricultural systems [7,14].

3. Temporal and Structural Effects

Hydrogel impacts are transient, with hydrological benefits peaking within weeks before diminishing. Microbial activity surges during hydrogel efficacy periods but stabilizes as hydration declines [7,110]. Biodegradability further modulates long-term effects: non-degradable PEGDMA avoids confounding metabolic measurements [109,111], while degradable variants introduce organic fragments that may stimulate secondary microbial activity [111]. Hydrogel porosity and permeability govern microbial dispersal, with 3D-bioprinted synthetic soil aggregates (SSAs) demonstrating how pore networks regulate resource access and community assembly [7,109].

These findings highlight synthetic hydrogels as dual tools for probing soil ecology and enhancing agricultural sustainability, though their application requires balancing short-term benefits against legacy impacts on microbial networks.

## 6. Challenges and Future Directions in Hydrogel Applications in Agriculture

Despite the numerous advantages that hydrogels offer in enhancing agricultural productivity, several challenges remain that hinder their widespread adoption. Addressing these challenges requires a multidisciplinary approach that integrates advancements in material science, environmental science, and agronomy. This section outlines the key challenges faced in the application of hydrogels in agriculture and explores future research directions that could unlock the full potential of these materials.

### 6.1. Economic Feasibility and Cost Considerations of Hydrogel Application in Agriculture

One of the main challenges to the widespread adoption of hydrogels in agriculture is their cost. High-quality hydrogels, especially those derived from synthetic polymers, are relatively expensive to produce, making them less accessible for small-scale farmers in developing regions [17]. While the long-term benefits—such as increased crop yield and reduced water and fertilizer use—may justify the initial investment, the upfront cost remains a significant barrier [7,15]. Additionally, the cost-effectiveness of hydrogels depends on several factors, including crop type, soil properties, and climate, which makes it difficult to generalize their economic benefits across diverse agricultural systems [7].

The profitability of hydrogel application in agriculture is influenced by whether the reduction in irrigation and fertilizer expenses outweighs the added cost of purchasing and applying hydrogels. Studies suggest that hydrogels can contribute to economic gains by improving water retention and nutrient efficiency, ultimately enhancing crop yields. For instance, a four-year field study on Indian mustard production found that hydrogel application could lead to a profit increase of 3.31 USD/acre/day (approximately 820 USD/km^2^/day) under deficit irrigation conditions [112]. However, comprehensive economic studies quantifying the net profitability of hydrogel use remain limited.

A techno-economic analysis estimated the production cost of a starch-based controlled-release fertilizer to be approximately 700 USD/ton, with a selling price of 900 USD/ton [113]. Applying hydrogels at a rate of 120 kg/acre (≈30,000 kg/km^2^) would add an extra cost of 108 USD/acre (≈27,000 USD/km^2^) to an agricultural system. While this expense could be offset by yield improvements, more studies are needed to assess these economic trade-offs comprehensively. Moreover, systematic economic evaluation methods, such as life cycle cost analysis (LCCA), are rarely applied to hydrogel usage in agriculture. LCCA, commonly used in engineering and energy systems, evaluates cost-effectiveness by considering both initial investment and future expenditures [15,114]. Implementing LCCA in future research could provide a clearer understanding of the long-term financial viability of hydrogels in different agricultural contexts.

To mitigate cost barriers, research should focus on developing affordable, sustainable hydrogel alternatives, particularly those derived from natural and renewable materials. Advances in biopolymer synthesis and the utilization of agricultural by-products may enable the production of cost-effective, biodegradable hydrogels that are both environmentally and economically viable [7,15]. Additionally, optimizing large-scale manufacturing processes and improving supply chain logistics could help reduce production costs, making hydrogels more accessible to a broader range of farmers worldwide.

### 6.2. Environmental Impact and Degradability

The environmental impact of synthetic hydrogels, particularly those that are not biodegradable, is another critical challenge. Many conventional hydrogels are derived from petrochemical sources and do not break down easily in the environment, raising concerns about soil health and long-term sustainability [16]. Over time, the accumulation of non-biodegradable hydrogel residues in the soil could potentially alter soil structure, affect microbial communities, and reduce soil fertility.

To mitigate these environmental concerns, there is a growing interest in developing biodegradable hydrogels made from natural polymers such as cellulose, chitosan, and starch [6]. These biodegradable alternatives decompose naturally in the soil, minimizing their environmental footprint. However, the challenge lies in ensuring that these hydrogels retain the desired properties, such as water retention and mechanical strength, while maintaining their biodegradability [62]. Future research should aim to optimize the balance between performance and sustainability, enabling the development of hydrogels that are both effective and environmentally benign.

### 6.3. Performance Variability Across Different Soil Types

The effectiveness of hydrogels can vary significantly depending on the soil type, climate, and crop species. For example, hydrogels that perform well in sandy soils with low water retention may not be as effective in clayey soils where waterlogging is a concern [10,84]. Similarly, the ionic strength and pH of the soil can influence the swelling and deswelling behavior of hydrogels, affecting their ability to retain and release water [62]. This variability poses a challenge for farmers who need reliable solutions that work consistently across different environmental conditions.

Addressing this challenge requires a better understanding of the interactions between hydrogels and various soil types. Research should focus on developing soil-specific hydrogels that are tailored to the unique properties of different soils, such as their texture, mineral content, and microbial activity [19]. Additionally, integrating hydrogels with soil amendments, such as organic matter or minerals, could enhance their performance and ensure that they provide consistent benefits across a wide range of agricultural environments. Table 7 highlights the challenges in hydrogel applications and suggests potential research directions.

### 6.4. Integration with Precision Agriculture

The future of hydrogel applications in agriculture lies in their integration with precision agriculture technologies. Precision agriculture involves the use of data-driven approaches to optimize farming practices, and hydrogels can play a crucial role in this context by providing targeted water and nutrient delivery [7,26]. For example, hydrogels can be combined with soil moisture sensors to create smart irrigation systems that adjust watering schedules based on real-time soil conditions. This integration can lead to more efficient water use, reduced waste, and higher crop yields.

However, integrating hydrogels with precision agriculture technologies presents several challenges. These include the need for sensors that can accurately measure the moisture content within hydrogel-treated soils and the development of algorithms that can interpret these data to optimize irrigation [6]. Additionally, the cost of these technologies may be prohibitive for smallholder farmers, necessitating research into affordable and scalable solutions that can be widely adopted.

### 6.5. Policy and Regulatory Considerations

The adoption of hydrogels in agriculture is also influenced by policy and regulatory frameworks. In many regions, there are limited regulations governing the use of synthetic polymers in agriculture, which can lead to concerns about environmental safety and product quality [19]. To promote the responsible use of hydrogels, governments and regulatory bodies should establish clear guidelines for their production, application, and disposal. These guidelines should prioritize sustainability and encourage the use of environmentally friendly materials.

In addition, policies that provide incentives for the adoption of hydrogels, such as subsidies or tax breaks for farmers who use biodegradable hydrogels, could help accelerate their uptake [23]. Collaborative efforts between governments, research institutions, and industry stakeholders are essential to developing a regulatory framework that supports innovation while protecting environmental and public health.

## 7. Case Studies and Real-World Applications of Hydrogels in Agriculture

Hydrogels have been widely tested and applied across various agricultural settings, demonstrating their potential to address water scarcity, improve crop yields, and enhance soil health. This section presents a series of case studies from different regions and agricultural systems, highlighting the practical benefits and challenges of using hydrogels in real-world applications.

### 7.1. Case Study: Improving Drought Resilience in Maize Cultivation in Sub-Saharan Africa

In Sub-Saharan Africa, where drought is a persistent challenge, the application of hydrogels in maize cultivation has shown promising results. In a study conducted in Kenya, researchers applied superabsorbent hydrogels to maize fields to assess their impact on water retention and crop yield. The results demonstrated that fields treated with hydrogels experienced a 25% increase in soil moisture levels compared to untreated fields [17]. This increase in moisture translated into a 20% improvement in maize yield during the dry season, highlighting the potential of hydrogels to enhance food security in drought-prone regions.

The study also revealed that hydrogels reduced the need for frequent irrigation, allowing farmers to conserve water and reduce labor costs. However, the initial cost of the hydrogels was a significant barrier for small-scale farmers, indicating the need for more affordable alternatives or financial support mechanisms [15].

### 7.2. Case Study: Enhancing Crop Yields in Semi-Arid Regions of India

In India, hydrogels have been used to improve crop yields in semi-arid regions where water scarcity limits agricultural productivity. A case study conducted in the state of Rajasthan evaluated the effectiveness of hydrogels in the cultivation of pearl millet, a staple crop in the region. The application of hydrogels resulted in a 30% increase in grain yield, primarily due to improved water retention and availability during critical growth stages [6].

Additionally, the study found that hydrogel-treated fields required 40% less water than untreated fields, demonstrating the efficiency of hydrogels in water conservation. Farmers also reported improved soil structure and reduced soil erosion, further contributing to the sustainability of agricultural practices in the region [7]. The success of this case study has led to wider adoption of hydrogels in other semi-arid regions of India. Table 8 provides a summary of key case studies on hydrogel applications in agriculture.

### 7.3. Case Study: Sustainable Agriculture in Southern Europe

In Southern Europe, particularly in Spain and Italy, vineyards have adopted hydrogels to improve water use efficiency and grape quality. A study conducted in the Mediterranean region demonstrated that hydrogel application in vineyards increased grape yield by 15% and enhanced quality metrics like sugar content and flavor. The hydrogels maintained consistent soil moisture, mitigating irregular rainfall impacts and preventing water stress during critical ripening periods [119].

Furthermore, the use of hydrogels reduced the need for supplemental irrigation by 25%, contributing to water conservation in a region where water resources are increasingly scarce [120]. The success of this approach has prompted further research into the use of hydrogels in other high-value crops, such as olives and citrus fruits, in the Mediterranean region [19].

### 7.4. Case Study: Managing Soil Salinity and Drought in the Middle East

Hydrogels have emerged as a promising solution for addressing water scarcity and soil salinity challenges in agriculture, particularly in arid regions like the Middle East. These superabsorbent polymers can significantly enhance soil moisture retention and improve crop productivity under water-stressed conditions.

Polyacrylamide (PAM) hydrogels are widely used in agriculture for their ability to absorb and retain large amounts of water. When incorporated into soil, PAM hydrogels can absorb water up to 400–500 times their dry weight, gradually releasing it to maintain consistent soil moisture levels. Field studies have demonstrated that soils treated with hydrogels exhibit a 30–50% reduction in irrigation frequency, while also increasing crop yields by 15–20% in crops like maize [7,32,121].

The benefits of hydrogels extend beyond just water retention. They also play a crucial role in erosion control and soil stabilization by enhancing soil structure and reducing surface runoff. Laboratory simulations and field data have shown a 40% reduction in soil erosion in hydrogel-treated plots [60,69,101,121].

In saline soil conditions, which are common in the Middle East, hydrogels have shown the potential to mitigate salt stress. A study on maize cultivation in saline soils found that hydrogel application reduced sodium concentration in soil leachate from 1499 to 1219 mg L^–1^ at high salinity levels (4.5 dS m^–1^). However, the same study noted that excessive hydrogel application (0.07% *w*/*w*) could potentially increase soil sodium content and reduce potassium uptake by plants, highlighting the importance of proper application rates [122].

While hydrogels offer significant benefits, their adoption faces challenges in some regions. For instance, in Jordan, despite the availability of imported hydrogels, their use in farming remains limited due to factors such as lack of farmer awareness and government water subsidies that reduce incentives for water conservation [7,123,124,125].

As research continues, the potential of hydrogels to contribute to sustainable agriculture in water-stressed regions is becoming increasingly evident. However, further studies are needed to optimize application rates, assess long-term environmental impacts, and develop strategies to overcome adoption barriers in different agricultural contexts.

### 7.5. Future Prospects: Expanding Hydrogel Use in Global Agriculture

The success of hydrogel applications in various agricultural settings demonstrates their significant potential to address challenges across different regions and climates. Hydrogels offer promising solutions for water conservation, soil improvement, and crop productivity enhancement. However, their broader adoption depends on overcoming key barriers and advancing research in several areas.

Improving Cost-Effectiveness and Accessibility

One of the primary challenges in expanding hydrogel use is cost. While initial investments in hydrogel technology can be offset by long-term benefits, the upfront costs may deter some farmers, especially in developing regions. Research is ongoing to develop more cost-effective hydrogel formulations and application methods. For instance, studies are exploring the use of agricultural waste materials to create biodegradable hydrogels, potentially reducing production costs and improving sustainability [107]. The development of eco-friendly hydrogels with enhanced water retention capabilities under varying moisture conditions has been proposed to improve seedling growth, germination, and overall plant development [107].

Enhancing Environmental Compatibility

As hydrogel use expands, ensuring their environmental safety becomes crucial. Future research should focus on developing fully biodegradable hydrogels that leave no harmful residues in the soil. Recent advancements in eco-friendly hydrogels, such as those derived from cellulose and bamboo, show promise for sustainable agriculture applications [107]. However, concerns remain about the potential long-term environmental impacts of synthetic hydrogels. Studies have highlighted the need for systematic toxicity assessments of different hydrogel forms in the medium and long term to ensure their sustainability [3,11,24,81].

Tailoring Hydrogels to Specific Crops and Conditions

To maximize the benefits of hydrogels, research is needed to optimize formulations for specific crops and soil types. This customization could involve adjusting water retention capacities, nutrient release rates, and degradation timelines to match the needs of different agricultural systems. Studies have shown that hydrogels can be tailored to act as water reservoirs charged with nutrients for efficient delivery to plant roots, thereby optimizing water consumption and reducing irrigation frequency [107].

Integrating with Smart Farming Technologies

The future of hydrogel use in agriculture likely involves integration with smart farming technologies. For example, hydrogel-based sensors are being developed to monitor soil moisture levels and trigger irrigation systems, potentially leading to more precise water management [7,126]. These “smart” soil systems can capture water from the air to keep plants hydrated and manage the controlled release of fertilizer for a constant supply of nutrients [7,126].

Scaling Up and Knowledge Transfer

Expanding hydrogel use globally requires collaboration between governments, research institutions, and industry stakeholders. Initiatives to promote knowledge transfer and provide technical support to farmers will be crucial. Case studies from various regions, such as the Middle East and Sub-Saharan Africa, where hydrogels have shown significant water savings and yield improvements, can serve as models for broader implementation [7].

Addressing Regional Challenges

Different regions face unique agricultural challenges that hydrogels could help address. For instance, in Sub-Saharan Africa, where water scarcity and poor soil quality are major issues, hydrogels could significantly improve soil moisture retention and boost crop yields. Similarly, in Latin America and Australia, hydrogel technology could help stabilize production in drought-prone areas [126].

Regulatory Frameworks and Standards

As hydrogel use expands, developing appropriate regulatory frameworks and quality standards will be essential. This will ensure the safe and effective use of hydrogels in agriculture while promoting innovation and market growth. Policymakers should consider the potential environmental impacts and sustainability of different hydrogel formulations when developing regulations [123,124,125].

Long-term Impact Studies

While the short-term benefits of hydrogels are well-documented, more research is needed on their long-term impacts on soil health, microbial activity, and crop productivity. Such studies will be crucial for understanding the sustainability of hydrogel use in agriculture over extended periods. Researchers have emphasized the need for longer field tests and integration of different types of fertilizers to fully assess the potential of hydrogel technology [7].

The future of hydrogels in global agriculture is promising, with the potential to significantly contribute to sustainable farming practices. By addressing current limitations and leveraging ongoing research and innovation, hydrogels could play a transformative role in making agriculture more resilient to climate change and resource scarcity. The key to realizing this potential lies in continued research, collaborative efforts, and strategic implementation across diverse agricultural landscapes.

## 8. Comparative Analysis of Hydrogel Types and Their Suitability for Different Agricultural Practices

Hydrogels are classified into various types based on their chemical composition, source (natural vs. synthetic), and crosslinking mechanism. Each type of hydrogel has distinct properties that make it more suitable for certain agricultural applications than others. Understanding these differences is essential for selecting the appropriate hydrogel for specific crops, soil types, and environmental conditions. This section provides a comparative analysis of different hydrogel types and their suitability for various agricultural practices.

### 8.1. Natural vs. Synthetic Hydrogels

Natural hydrogels, derived from biopolymers such as cellulose, starch, and chitosan, are gaining popularity due to their biodegradability and minimal environmental impact. These hydrogels are particularly valuable in sustainable agricultural practices where environmental conservation is a priority [6]. However, they often have lower mechanical strength and water retention capacity compared to synthetic hydrogels, which can limit their effectiveness in certain applications.

Synthetic hydrogels, conversely, are made from synthetic polymers such as polyacrylamide (PAM), polyvinyl alcohol (PVA), and polyethylene glycol (PEG). These hydrogels are engineered to have high water absorption capacity and durability, making them ideal for industrial agriculture where consistent performance is required [34,49,66]. However, their non-biodegradable nature raises concerns about long-term environmental impact, prompting ongoing research into creating biodegradable synthetic hydrogels that combine the advantages of both natural and synthetic materials [15]. Table 9 presents a comparative analysis of natural and synthetic hydrogels.

### 8.2. Reversible vs. Irreversible Hydrogels

Hydrogels can also be categorized based on the nature of their crosslinking, which significantly influences their structural stability and functional applications. Reversible hydrogels, also known as physically crosslinked hydrogels, are held together by non-covalent interactions such as hydrogen bonds, ionic forces, or hydrophobic associations. These interactions enable them to undergo phase transitions in response to environmental changes, such as fluctuations in temperature, pH, or moisture levels [19]. Due to their dynamic and responsive behavior, reversible hydrogels are widely utilized in applications requiring controlled water release, such as precision irrigation systems and moisture-sensitive agricultural formulations. However, their relatively lower mechanical strength compared to irreversible hydrogels may limit their effectiveness in more demanding agricultural environments.

In contrast, irreversible hydrogels are chemically crosslinked through covalent bonds, forming a stable and permanent polymer network. This structural integrity allows them to maintain their properties under extreme conditions, including high temperatures, salinity, and prolonged exposure to environmental stressors [7,124]. As shown in Table 10, irreversible hydrogels exhibit superior mechanical strength and long-term durability, making them particularly suitable for applications such as soil conditioners in arid and saline regions. Their ability to retain water and withstand harsh conditions enhances soil moisture retention and promotes sustainable agricultural practices in challenging climates.

Overall, Table 10 provides a comparative analysis of reversible and irreversible hydrogels, highlighting their distinct advantages and limitations. While reversible hydrogels offer flexibility and responsiveness, irreversible hydrogels provide enhanced stability and durability. Understanding these differences is crucial for selecting the appropriate hydrogel type for specific agricultural applications, ensuring optimal performance based on environmental conditions and crop requirements.

### 8.3. Soil-Specific Hydrogel Applications

The efficiency of hydrogels in agricultural applications is highly dependent on soil type, climate conditions, and crop species. In sandy soils, which exhibit low water retention capacity and rapid drainage, hydrogels play a crucial role in enhancing moisture availability by absorbing and retaining substantial amounts of water [17]. This property helps mitigate water loss and supports plant growth in arid and semi-arid regions. Conversely, in clayey soils, where poor drainage often leads to waterlogging, hydrogels with controlled absorption and release mechanisms are essential for maintaining optimal soil moisture levels while preventing excess water accumulation.

The performance of hydrogels is also influenced by soil chemical properties, particularly ionic strength and pH. In saline soils, for instance, high ion concentrations can interfere with hydrogel swelling, thereby reducing their water retention efficiency [6]. To overcome such limitations, researchers have developed soil-specific hydrogels engineered to function effectively under diverse soil conditions. These specialized formulations are designed to optimize hydrogel performance by considering key factors such as soil texture, mineral composition, and microbial interactions [19]. As outlined in Table 11, the suitability of different hydrogel types varies based on soil characteristics, emphasizing the importance of selecting the appropriate hydrogel formulation for specific agricultural environments. Understanding these interactions allows for the strategic application of hydrogels, ensuring maximum efficiency in water retention, soil structure improvement, and overall plant health.

### 8.4. Hybrid Hydrogels: Combining Strengths

Hybrid hydrogels, which combine natural and synthetic polymers, represent an emerging area of research. These hydrogels aim to balance the biodegradability of natural hydrogels with the superior mechanical strength and water retention capacity of synthetic hydrogels [15]. Hybrid hydrogels are particularly promising for precision agriculture, where tailored solutions are required to meet specific crop and soil needs while minimizing environmental impact.

For example, a hybrid hydrogel composed of chitosan and polyacrylamide has shown enhanced water retention and biodegradability, making it suitable for both short-term and long-term applications [129]. These hydrogels can be engineered to release water and nutrients in response to specific environmental triggers, offering greater control over agricultural inputs and improving overall efficiency.

## 9. Innovations and Future Developments in Hydrogel Technology for Agriculture

The field of hydrogel technology is rapidly evolving, driven by the need to address the challenges of modern agriculture, including water scarcity, soil degradation, and climate change. Recent innovations in hydrogel design and application are expanding the potential of these materials to support sustainable agricultural practices. This section explores some of the most promising developments in hydrogel technology and their potential impact on agriculture.

### 9.1. Smart Hydrogels for Precision Agriculture

Smart hydrogels represent a significant advancement in agricultural resource management, offering dynamic responses to environmental triggers like temperature, pH, and moisture levels. These materials enable precise, on-demand delivery of water and nutrients, aligning with precision agriculture principles to optimize inputs and minimize waste.

Temperature-responsive hydrogels have shown promise in mitigating heat stress in crops. Researchers have developed hydrogels that absorb moisture at night and release it during daytime heat, reducing irrigation needs while improving plant growth [107]. This technology is particularly beneficial for crops requiring stable root-zone conditions.

pH-sensitive hydrogels offer targeted nutrient delivery based on soil acidity or alkalinity. Hydrogels loaded with NPK fertilizers can release nutrients in response to soil pH changes, improving nutrient-use efficiency and reducing fertilizer leaching [131]. This precision in nutrient release enhances crop yields while minimizing environmental impact.

Moisture-responsive hydrogels act as soil moisture sensors, releasing water only when soil water potential drops below critical thresholds. These hydrogels can significantly enhance water retention in agricultural systems, enabling substantial water savings compared to traditional irrigation methods [98].

The integration of smart hydrogels with digital agriculture technologies is opening new frontiers in farm management. Hydrogel-based soil sensors can transmit real-time data to automated irrigation systems, enabling closed-loop control of resource application [132].

Sustainability considerations are driving innovations in hydrogel composition. Biodegradable hydrogels derived from natural materials like cellulose and agro-waste are reducing reliance on synthetic polymers, addressing concerns about soil contamination [7,133].

Despite these promising developments, challenges remain in scaling up smart hydrogel technologies. Production costs for advanced hydrogels are still higher than conventional polymers, limiting their adoption in some contexts. Additionally, long-term field studies are needed to fully assess the impacts of hydrogels on soil ecosystems [134].

Looking ahead, researchers are exploring multi-stimuli responsive hydrogels that can concurrently adapt to changes in temperature, pH, and ionic strength—a crucial development for managing crops in diverse soil conditions [135]. Table 12 provides an overview of recent smart hydrogel innovations and their potential applications in agriculture, highlighting advancements in water retention, nutrient delivery, and environmental responsiveness.

### 9.2. Biodegradable Hydrogels for Sustainable Agriculture

As environmental concerns continue to grow, the development of biodegradable hydrogels has become a major focus of research. These hydrogels are made from natural polymers such as cellulose, chitosan, and starch, which decompose naturally in the soil without leaving harmful residues [6]. Biodegradable hydrogels offer a sustainable alternative to conventional synthetic hydrogels, which can persist in the environment and potentially harm soil health over time.

Recent innovations in the formulation of biodegradable hydrogels have improved their water retention capacity, mechanical strength, and degradation rate, making them more suitable for a wider range of agricultural applications [23]. For example, researchers have developed a starch-based hydrogel that retains water as effectively as synthetic hydrogels while degrading completely within a growing season. This innovation has the potential to make sustainable hydrogel use more accessible to farmers, particularly those in developing regions.

### 9.3. Nanocomposite Hydrogels for Enhanced Performance

Nanocomposite hydrogels, which incorporate nanoparticles into the polymer network, represent another innovative development in hydrogel technology. The addition of nanoparticles can significantly enhance the properties of hydrogels, such as their mechanical strength, water retention capacity, and responsiveness to environmental stimuli [19]. For instance, hydrogels reinforced with silica nanoparticles have been shown to exhibit superior durability and water retention in harsh soil conditions compared to conventional hydrogels [16].

Nanocomposite hydrogels are particularly promising for use in challenging agricultural environments, such as saline soils or areas prone to extreme temperatures. Their enhanced properties allow them to provide more consistent and reliable performance, supporting crop growth and resilience in the face of environmental stressors. Table 13 summarizes recent developments in nanocomposite hydrogels and their specific applications in agriculture, highlighting advancements in water retention, controlled nutrient release, and soil improvement for enhanced crop productivity.

### 9.4. Multi-Functional Hydrogels

Multi-functional hydrogels represent a significant advancement in agricultural technology, offering integrated solutions to address multiple challenges simultaneously. These innovative materials combine water retention, nutrient delivery, and pest control capabilities within a single system, potentially revolutionizing sustainable farming practices.

Recent research has demonstrated the efficacy of multi-functional hydrogels in enhancing water use efficiency and nutrient management. For instance, hydrogel–fertilizer blends have shown the ability to reduce irrigation frequency by 35–50% while improving nitrogen-use efficiency by 40% compared to conventional fertilizers [130]. These systems utilize ionic crosslinking to bind NPK fertilizers within the polymer network, enabling controlled nutrient release in response to soil moisture levels.

The integration of biological agents into hydrogel matrices has opened new avenues for pest and disease management. Studies have shown that hydrogels incorporating beneficial microbes can significantly reduce fungal pathogens while promoting plant growth. For example, carboxymethyl chitosan–alginate hydrogels loaded with Ensifer C5 rhizobacteria increased rapeseed yields by 30% under field conditions while suppressing Fusarium wilt through induced systemic resistance [7].

Environmental adaptability is another key feature of multi-functional hydrogels. Formulations have been developed to address specific soil conditions, such as calcium–alginate hydrogels that buffer pH fluctuations in acidic soils while maintaining high phosphorus availability [6]. Similarly, sulfonated cellulose hydrogels have shown promise in mitigating salinity stress by sequestering sodium ions from the root zone [134].

While the potential of multi-functional hydrogels is significant, challenges remain in scaling up production and reducing costs. Current research is focused on developing biodegradable formulations derived from agricultural waste materials, which could improve sustainability and reduce production costs [39,62,88,111,130].

As research continues to advance, multi-functional hydrogels are poised to play a crucial role in the future of sustainable agriculture, particularly in integrated pest management and organic farming systems. Their ability to simultaneously address water scarcity, nutrient deficiencies, and pest control offers a holistic approach to crop management that aligns with the goals of precision agriculture and environmental stewardship.

### 9.5. Future Directions and Research Priorities

The continued development of hydrogel technology will require interdisciplinary collaboration between material scientists, agronomists, and environmental scientists. Key research priorities include the following:

1. Developing Cost-Effective Biodegradable Hydrogels: reducing the cost of biodegradable hydrogels to make them accessible to smallholder farmers, particularly in developing regions [6].
2. Enhancing the Performance of Smart Hydrogels: improving the responsiveness and reliability of smart hydrogels to ensure consistent performance in varying environmental conditions [15].
3. Exploring New Nanocomposite Materials: investigating the use of novel nanoparticles to enhance the properties of hydrogels, particularly for use in extreme environments [19].
4. Integrating Multi-Functional Hydrogels with Precision Agriculture: developing multi-functional hydrogels that can be seamlessly integrated into precision agriculture systems, enhancing overall farm efficiency and sustainability [88].

## 10. Economic and Environmental Implications of Hydrogel Use in Agriculture

The use of hydrogels in agriculture offers both economic benefits and environmental challenges. Understanding these implications is crucial for evaluating the overall sustainability and feasibility of hydrogel-based solutions in farming. This section provides an in-depth analysis of the economic and environmental impacts of hydrogel use, highlighting both the advantages and potential drawbacks.

### 10.1. Economic Benefits of Hydrogel Use

Hydrogels can offer significant economic advantages, particularly in water-scarce regions where irrigation costs are high. By improving soil moisture retention, hydrogels reduce the frequency of irrigation, leading to lower water usage and reduced labor costs [136]. This is especially beneficial in regions where water is a limited and expensive resource. Additionally, hydrogels can enhance crop yields by providing consistent moisture and nutrients, leading to higher productivity and increased farm income [15].

In terms of crop quality, hydrogels can improve the market value of produce by enhancing fruit size, sugar content, and overall nutritional quality. For instance, in grape cultivation, the use of hydrogels has been shown to improve grape quality, leading to better wine production and higher market prices [128]. These benefits can offset the initial cost of hydrogel application, particularly for high-value crops.

### 10.2. Cost Considerations and Barriers to Adoption

Despite the economic benefits, the high initial cost of hydrogels remains a significant barrier to widespread adoption, especially for smallholder farmers in developing regions. Synthetic hydrogels, which are often more effective in water retention, tend to be more expensive due to their complex production processes [6]. Although biodegradable hydrogels offer a more environmentally friendly alternative, they are also costly to produce, further limiting their accessibility [23].

For many farmers, the upfront investment in hydrogels is difficult to justify, particularly when the economic return is uncertain or depends on factors such as crop type, soil conditions, and local climate. To address this challenge, there is a need for government subsidies, financial incentives, or cost-sharing programs that can reduce the financial burden on farmers and encourage the adoption of hydrogel technologies [19].

### 10.3. Environmental Benefits

Hydrogels offer several environmental benefits that contribute to the sustainability of agricultural practices. By improving soil moisture retention, hydrogels reduce the need for frequent irrigation, conserving water resources and reducing the energy required for water pumping and distribution [129]. This is particularly important in arid and semi-arid regions where water scarcity is a major constraint to agricultural productivity.

Moreover, hydrogels can help mitigate soil erosion by stabilizing the soil structure and reducing surface runoff. In sloped or degraded lands, the use of hydrogels has been shown to improve soil retention, preventing the loss of valuable topsoil and nutrients [16]. These environmental benefits make hydrogels an attractive option for sustainable agriculture, especially in regions facing environmental degradation. Table 14 outlines the environmental benefits of hydrogel use.

### 10.4. Environmental Challenges and Risks

Despite their benefits, the environmental impact of hydrogels, particularly synthetic ones, cannot be overlooked. Non-biodegradable synthetic hydrogels can persist in the soil for extended periods, potentially leading to soil contamination and disruption of soil ecosystems [15]. Over time, these materials may break down into microplastics, which pose a risk to soil health and the broader environment [137].

Biodegradable hydrogels, while environmentally friendly, also present challenges. Their degradation rate can be influenced by soil conditions, temperature, and microbial activity, which can lead to inconsistent performance in different environments [23,56]. Additionally, the production of biodegradable hydrogels often requires the use of natural resources, raising concerns about the sustainability of large-scale production.

To mitigate these environmental risks, future research should focus on developing hydrogels that balance performance with sustainability. This includes exploring new materials and formulations that enhance biodegradability without compromising water retention capacity or mechanical strength [19].

### 10.5. Life Cycle Assessment of Hydrogel Use

A comprehensive life cycle assessment (LCA) of hydrogel use in agriculture is essential to fully understand their economic and environmental impacts. LCA considers all stages of a product’s life cycle, from raw material extraction and production to use and disposal [6,7]. For hydrogels, this includes the energy and resources required for production, the environmental impact of their use in agriculture, and the long-term effects of their degradation or accumulation in the soil.

Preliminary LCA studies suggest that while hydrogels can reduce water use and improve crop yields, the environmental costs associated with their production and disposal must be carefully managed. This highlights the need for continuous innovation in hydrogel technology to improve sustainability outcomes while maximizing agricultural benefits [16].

## 11. Conclusions and Future Outlook

The application of hydrogels in agriculture represents a significant advancement in the quest for sustainable and efficient farming practices. As global challenges such as water scarcity, soil degradation, and climate change continue to threaten agricultural productivity, hydrogels offer a promising solution to mitigate these issues. Through their ability to retain water, deliver nutrients, and improve soil structure, hydrogels have proven effective in enhancing crop resilience, increasing yields, and conserving vital resources. Despite the promising benefits of hydrogels in agriculture, certain limitations need to be addressed for their widespread adoption. Hydrogel performance varies depending on soil texture, climate conditions, and polymer characteristics, leading to inconsistencies in field results. Furthermore, the long-term environmental impact of synthetic hydrogels, particularly their persistence and effects on soil microbial communities, remains a concern. Future research should focus on field-scale evaluations across diverse agro-ecological zones, as well as the development of biodegradable alternatives to minimize potential environmental risks. A deeper understanding of cost-effectiveness and sustainability will also aid in the practical implementation of hydrogels in modern agriculture.

### 11.1. Summary of Key Findings

Throughout this review, several key benefits and challenges associated with hydrogel use in agriculture have been highlighted:

1. Water Conservation and Efficiency: Hydrogels significantly reduce the need for frequent irrigation, making them invaluable in drought-prone regions. By retaining moisture in the soil, they ensure a consistent water supply to plants, leading to improved crop yields and reduced water usage [7,15,129].
2. Enhanced Crop Growth and Resilience: The ability of hydrogels to improve root development, reduce nutrient leaching, and provide a stable growing environment leads to better plant health and higher productivity. This is particularly important in regions facing environmental stressors such as salinity and temperature extremes [6,128].
3. Environmental Benefits and Challenges: While hydrogels offer numerous environmental benefits, including soil stabilization and reduced erosion, their long-term impact on soil health, particularly with non-biodegradable synthetic hydrogels, remains a concern. Continued innovation in biodegradable hydrogel technology is crucial for minimizing environmental risks [17,23,40,59,78].
4. Economic Implications: Although hydrogels provide significant economic benefits by increasing yields and reducing water and fertilizer costs, the high initial investment remains a barrier for many farmers. Cost reduction strategies and financial support mechanisms are needed to make hydrogel technology accessible to a broader range of farmers [7,19].

### 11.2. Future Outlook

To ensure the broader adoption of hydrogels in agriculture, future research should focus on enhancing their economic viability, optimizing their environmental impact, and expanding their applicability to various agroecosystems. One key avenue is the development of biodegradable hydrogel formulations that reduce long-term soil accumulation issues. Emerging materials such as starch-, cellulose-, and chitosan-based hydrogels show promise for combining sustainability with water retention efficiency [7]. Additionally, smart hydrogels capable of responding to soil moisture levels and temperature changes have the potential to revolutionize precision agriculture by optimizing irrigation efficiency [37].

Beyond material advancements, efforts should be made to scale up commercial production and reduce costs, making hydrogel technology more accessible, particularly for smallholder farmers. Government incentives, public–private partnerships, and supply chain optimizations could drive down production costs, facilitating widespread adoption [113]. Furthermore, long-term field trials and regulatory frameworks should be established to assess the ecological impact of synthetic hydrogels on soil health and microbial communities [112]. Addressing these aspects will ensure that hydrogels continue to evolve as a commercially viable and environmentally sustainable solution for modern agriculture. Several areas hold particular promise for the advancement of hydrogels in agriculture:

1. Development of Cost-Effective Biodegradable Hydrogels: Research into natural and renewable materials for hydrogel production could lead to more affordable and environmentally friendly alternatives to current synthetic hydrogels. This would make hydrogels accessible to smallholder farmers, particularly in developing regions [6].
2. Integration with Precision Agriculture: The synergy between hydrogels and precision agriculture technologies offers significant potential for optimizing resource use and improving crop management. Smart hydrogels that respond to environmental stimuli, combined with data-driven farming practices, could revolutionize agricultural efficiency [23,25,26].
3. Expansion to New Crops and Regions: As the benefits of hydrogels become more widely recognized, their use is likely to expand beyond traditional applications. Future research should explore the potential of hydrogels in a wider range of crops and environmental conditions, including specialty crops and regions with extreme climates [16].
4. Addressing Environmental and Regulatory Challenges: To ensure the sustainable adoption of hydrogels, it is essential to address environmental concerns related to their long-term impact on soil health. Policymakers and industry stakeholders must work together to develop regulations that promote safe and responsible hydrogel use while encouraging innovation [7,24,36].

### 11.3. Final Thoughts

Hydrogels have the potential to play a transformative role in global agriculture, helping to meet the growing demand for food in an increasingly resource-constrained world. As the technology continues to evolve, the successful integration of hydrogels into agricultural practices will depend on a collaborative approach involving researchers, farmers, policymakers, and industry leaders. By addressing the challenges and capitalizing on the opportunities presented by hydrogel technology, we can create a more sustainable and resilient agricultural system for future generations.

## Figures and Tables

**Table 1:** Classification of Hydrogels by Origin.

Classification	Examples	Key Characteristics	Applications	References
Natural Hydrogels	Cellulose, Chitosan, Alginate, Collagen	Biodegradable, biocompatible, derived from polysaccharides/proteins	Biomedical (drug delivery, wound healing, tissue engineering)	[44,45,46]
Synthetic Hydrogels	Polyacrylamide (PAM), Polyvinyl Alcohol (PVA), Polyethylene Glycol (PEG)	High water absorption, durable, non-biodegradable	Industrial applications, controlled release systems	[45,47,48]
Hybrid/Composite Hydrogels	Gelatin–Alginate, Chitosan–Hyaluronic Acid, Collagen–Chondroitin Sulfate	Tailored properties (e.g., antimicrobial, stimuli-responsive), combine natural and synthetic polymers	Biomedical (drug delivery, tissue regeneration), smart materials	[49,50]

**Table 3:** Factors Influencing Hydrogel Water Retention and Release.

Factor	Mechanism of Influence	References
Crosslinking agents	Modify hydrogel network structure, affecting swelling capacity and stability	[60,76]
Polymer concentration	Higher concentrations (e.g., PAM ratios) enhance swelling ratio (SR) and water retention	[60]
pH sensitivity	Swelling capacity varies with pH due to ionizable groups (e.g., carboxylates)	[76,77]
Hydrogel application rate	Optimal rates (e.g., 0.3–0.6%) improve soil water-holding capacity without yield loss	[9,78]
Soil texture	Sandy soils show greater water retention improvements compared to clay soils	[9,76]
Additives (e.g., salts)	Hydratable salts enhance water retention by increasing osmotic pressure	[77]
Organic amendments	Combine with hydrogels to improve soil porosity and moisture retention	[78]
Mechanical strength	Crosslinked hydrogels resist degradation under stress, maintaining structural integrity	[60]
Biodegradability	Natural hydrogels (e.g., guar gum) degrade over time, affecting long-term retention	[6,79]
Reusability	Synthetic hydrogels retain functionality after repeated drying/wetting cycles	[60,76]
Temperature	Phase transition in temperature-responsive hydrogels regulates swelling and water release.	[62]

**Table 4:** Impact of Hydrogels on Soil Moisture Retention Across Different Soil Types.

Soil Type	Soil Texture Characteristics	Key Challenges	Hydrogel Mechanisms	Practical Benefits	Increase in Soil Moisture Retention	Optimal Application Rate	References
Sandy Soil	Large pores, rapid water infiltration	Low water retention, rapid drainage	Hydrogels fill pore spaces, reduce deep percolation, and increase water-holding capacity	Reduces irrigation frequency by 30%, improves crop yield in arid regions	35–50%	0.3–0.6%	[84]
Loamy Soil	Balanced pore structure, moderate retention	Variable moisture availability, uneven root growth	Enhances water availability, stabilizes moisture distribution, improves soil aggregation	Supports uniform root development, reduces drought stress in temperate climates	25–40%	0.2–0.4%	[84]
Clayey Soil	Small pores, poor drainage	Waterlogging, reduced aeration	Hydrogels prevent waterlogging by regulating moisture release, reduce compaction	Maintains optimal aeration, prevents root rot in waterlogged conditions	20–30%	0.1–0.3%	[85]
Saline Soil	High salt content, low water availability	Salt stress, reduced plant growth	Hydrogels improve water availability, reduce salt stress via osmotic adjustment	Enhances salt tolerance in crops, suitable for arid/saline regions	15–25%	0.2–0.5%	[86,87]

**Table 5:** Benefits of Hydrogel Seed Coating Across Different Crops.

Crop Type	Mechanisms of Action	Benefits of Hydrogel Coating	Examples of Improved Traits	Practical Benefits	References
Cereals	Water retention: hydrogels absorb and release moisture gradually, reducing drought stress. Nutrient delivery: encapsulated nutrients (e.g., N, P, K) enhance early root development.	Enhanced germination, improved drought tolerance	Wheat, Maize, Rice	Reduces irrigation frequency by 30%, increases yield in arid regions.	[6,7]
Legumes	Microbial encapsulation: alginate-based coatings protect beneficial microbes (e.g., Rhizobia), improving nitrogen fixation. Moisture regulation: prevents desiccation during germination.	Better seedling vigor, increased nutrient uptake	Soybean, Lentils, Peas	Enhances symbiotic nitrogen fixation, reduces fertilizer dependency.	[7,15]
Vegetables	Pathogen resistance: hydrogels create a physical barrier against soil-borne diseases (e.g., Fusarium). Moisture buffering: maintains consistent soil moisture for root growth.	Faster emergence, reduced susceptibility to soil-borne diseases	Tomatoes, Carrots, Onions	Reduces disease incidence by 40%, improves transplant survival in nurseries.	[9,92,93,94]
Oilseeds	Osmotic adjustment: hydrogels mitigate salt stress by balancing ion uptake. Seedling survival: retains moisture in arid soils, ensuring post-germination growth.	Improved moisture retention, enhanced seedling survival	Sunflower, Canola, Soybean	Increases drought resilience, reduces yield loss in saline soils.	[83]
Forage Crops	Root zone hydration: hydrogels maintain moisture in shallow root systems, critical for drought-prone pastures. Germination synchronization: ensures uniform emergence.	Increased germination rate, better drought resilience	Alfalfa, Clover, Ryegrass	Extends grazing seasons, improves pasture productivity in water-scarce regions.	[7]
Fiber Crops	Root development: hydrogels improve soil aeration and nutrient availability, promoting deep root growth. Seedling establishment: reduces transplant shock.	Enhanced root development, improved seedling establishment	Cotton, Jute, Flax	Increases fiber quality, reduces irrigation needs in industrial crop cultivation.	[82]
Fruit Crops	Early growth stimulation: hydrogels deliver nutrients (e.g., Ca, Mg) critical for fruit set. Drought resilience: maintains moisture during flowering/fruiting stages.	Higher seedling survival, improved early growth	Watermelon, Cucumber, Strawberries	Enhances fruit size and sugar content, reduces yield gaps in water-stressed conditions.	[7]

**Table 6:** Impact of Hydrogels on Root Development Across Different Crops.

Crop Type	Improved Root Traits	Specific Hydrogel Impact	Resulting Plant Benefits	Contextual Application Insights	References
Maize	Increased root length, higher root density	Enhanced water retention, deeper root growth	Improved drought resistance, higher biomass	Suitable for arid regions	[15]
Wheat	Deeper root penetration, greater root biomass	Better water and nutrient absorption	Higher yields, improved resilience to dry spells	Beneficial for semi-arid regions	[84]
Soybean	Enhanced lateral root growth, increased root volume	Improved soil moisture around root zone	Better nutrient uptake, increased nodulation	Supports nitrogen fixation in legumes	[6]
Tomato	Improved root structure, better water and nutrient uptake	Reduced soil compaction, stronger root anchorage	Higher fruit yield, improved fruit quality	Ideal for greenhouse and field cultivation	[86,90]
Cotton	More extensive root system, deeper soil exploration	Enhanced soil moisture retention	Increased fiber yield, better quality	Effective in both dryland and irrigated systems	[95,96]
Rice	Improved root mass, stronger root anchorage	Better water retention in paddy fields	Higher grain yield, reduced lodging	Crucial for flood-prone areas	[97,98,99]
Potato	Increased root and tuber formation, improved root spread	Enhanced water and nutrient delivery to tubers	Higher tuber yield, improved size consistency	Beneficial for commercial potato farming	[100,101]
Sunflower	Deeper taproot development, increased lateral roots	Improved water uptake during critical growth stages	Higher seed yield, better oil content	Particularly useful in dryland agriculture	[19,102]
Lettuce	Improved root system uniformity, better moisture retention	Enhanced growth in sandy soils	Higher leaf biomass, better marketability	Ideal for high-value leafy greens	[16,94]

**Table 7:** Challenges in Hydrogel Applications and Potential Research Directions.

Challenge	Description	Potential Research Direction	Citation
High Cost	Expensive production limits accessibility for small-scale farmers	Development of affordable, biodegradable hydrogels from natural resources	[17]
Environmental Impact	Non-biodegradable hydrogels raise concerns about soil health	Optimization of biodegradable hydrogels with comparable performance	[16]
Performance Variability	Inconsistent effectiveness across different soil types and climates	Design of soil-specific hydrogels tailored to local conditions	[7]
Degradability vs. Performance	Balancing biodegradability with mechanical strength and water retention	Research on hybrid hydrogels combining synthetic and natural polymers	[23,60]

**Table 8:** Summary of Key Case Studies on Hydrogel Applications in Agriculture.

Region	Crop Type	Hydrogel Benefits	Yield Improvement	Water Savings	Citation
Sub-Saharan Africa	Maize	Improved drought resilience, higher soil moisture retention, reduced irrigation frequency	20% increase	Reduced irrigation frequency	[7,115]
Semi-Arid India	Pearl Millet	Enhanced water retention, better soil structure, prolonged root hydration	30% increase	40% less water usage	[7,116]
Southern Europe	Grapes	Consistent moisture during berry development, improved fruit quality	15% increase	25% less water usage	[117]
Middle East	Tomato	Better fruit quality, reduced soil salinity, enhanced nutrient uptake	18% increase	30% less water usage	[118]
North America	Strawberries	Improved fruit firmness, longer shelf life, reduced irrigation needs	12% increase	20% less water usage	[117]

**Table 9:** Comparative Analysis of Natural and Synthetic Hydrogels.

Property	Natural Hydrogels	Synthetic Hydrogels	Best Applications	References
Raw Materials	Derived from natural sources like starch, chitosan, cellulose, lignin	Typically made from polyacrylamide, polyacrylate	-	[68,127]
Water Retention Capacity	Can increase soil water retention by 400% at 0.7% *w*/*w* concentration	Effective at increasing water retention at 0.1–1% *w*/*w* concentration	Drought-prone regions, High-intensity farming	[14,127]
Effect on Soil Hydraulic Conductivity	Decreased saturated hydraulic conductivity by 45–60% in sandy soils	Decreased saturated hydraulic conductivity in most cases, up to 90% reduction reported	To enhance soil hydraulic conductivity	[14]
Biodegradability	Higher biodegradability, shorter decomposition time	Lower biodegradability, longer persistence in soil	Organic farming, Environmental restoration	[68,111]
Environmental Impact	Generally considered more environmentally friendly	Concerns about long-term accumulation in soil	Sustainable agriculture	[68,111]
Cost	Lower cost of preparation	Higher production costs	High-value crops, Precision agriculture	[68]
Mechanical Strength	Often have lower mechanical resistance	Generally higher mechanical strength	Industrial agriculture, Long-term soil amendments	[127]
Crop Yield Improvement	Similar yield improvements observed	Similar yield improvements observed	To increase yield	[68]
Applications	Soil water retention, controlled nutrient release, erosion control	Soil water retention, controlled nutrient release, erosion control, heavy metal remediation	To improve agricultural production	[111,127]

**Table 10:** Comparative Analysis of Reversible and Irreversible Hydrogels.

Property	Reversible Hydrogels	Irreversible Hydrogels	Best Applications	References
Crosslinking Mechanism	Physical bonds (e.g., hydrogen bonds, ionic interactions, host–guest chemistry)	Covalent bonds (e.g., radical polymerization, click chemistry)	Reversible: responsive systems; Irreversible: permanent structural support	[19,38]
Environmental Responsiveness	Highly responsive to stimuli (pH, temperature, light, ionic strength)	Limited responsiveness; stable under most conditions	Reversible: smart nutrient delivery; Irreversible: static soil conditioning	[6,128]
Durability	Lower durability; reversible bonds break under stress	High durability; resistant to mechanical/chemical stress	Reversible: short-term use; Irreversible: long-term soil stabilization	[15,129]
Formation Mechanism	Non-covalent interactions enable reversible sol–gel transitions	Covalent crosslinking creates permanent networks	Reversible: controlled release systems; Irreversible: structural scaffolds	[19,38]
Stability	Less stable; prone to dissolution under changing conditions	Highly stable; retains structure in aqueous environments	Reversible: seasonal crops; Irreversible: perennial crops	[128]
Mechanical Strength	Moderate to low strength	High strength and elasticity	Irreversible: heavy clay soils; Reversible: sandy soils	[15]
Biodegradability	Typically biodegradable (e.g., alginate, chitosan)	Often non-biodegradable (e.g., polyacrylamide) unless modified	Reversible: organic farming; Irreversible: industrial agriculture	[6,129]
CO_2_ Sequestration	Limited due to reversible bonds	Higher potential due to long-term stability	Irreversible: carbon capture in soils	[19]
Suitability for Urban Farming	Ideal for short-term, responsive systems (e.g., vertical farms)	Better for long-term soil amendments (e.g., rooftop gardens)	Reversible: hydroponics; Irreversible: green infrastructure	[15,128]
Biodegradability	Often more biodegradable	Can be less biodegradable, depending on composition	-	[61]
Applications in Agriculture	Soil water retention, controlled nutrient release	Long-term soil conditioning, erosion control		[61,130]

**Table 11:** Suitability of Hydrogels for Different Soil Types.

Soil Type	Challenges Addressed by Hydrogels	Optimal Hydrogel Type	Application Examples	Citation
Sandy Soil	Low water retention, rapid drainage	High-absorption synthetic hydrogels	Maize, Wheat cultivation in arid regions	[17,60]
Clayey Soil	Waterlogging, poor aeration	Reversible hydrogels with controlled release	Rice, Cotton in semi-arid regions	[6]
Loamy Soil	Balanced moisture, variable retention	Biodegradable natural hydrogels	Vegetables, Fruits in mixed farming systems	[84]
Saline Soil	Reduced swelling, high ionic strength	Irreversible hydrogels with high mechanical strength	Tomato, Grapes in coastal regions	[41]
Alkaline Soil	pH-induced swelling reduction	pH-responsive hydrogels	Peppers, Lettuce in alkaline soil environments	[7,19]

**Table 12:** Smart Hydrogel Innovations and Their Agricultural Applications.

Smart Hydrogel Type	Trigger Mechanism	Agricultural Application	Benefits	Citation
Temperature-Sensitive	Swelling/deswelling in response to temperature changes	Heat stress management in crops	Protects crops from high temperatures, conserves water	[15]
pH-Sensitive	Swelling/deswelling in response to soil pH	Targeted nutrient release in variable pH soils	Optimizes nutrient availability, reduces fertilizer use	[63,135]
Moisture-Sensitive	Swelling/deswelling in response to soil moisture	Precision irrigation in water-scarce regions	Reduces irrigation frequency, conserves water	[7,23]
Ion-Sensitive	Response to ionic concentration in soil	Salinity management in coastal or arid regions	Protects crops from salt stress, improves yield	[6]

**Table 13:** Nanocomposite Hydrogels and Their Agricultural Applications.

Nanocomposite Type	Key Properties	Agricultural Application	Benefits	Citation
Silica Nanocomposite	High mechanical strength, improved water retention	Drought management in arid regions	Provides reliable water retention, enhances crop resilience	[16]
Carbon Nanotube-Based	Enhanced electrical conductivity, responsiveness	Precision agriculture, smart farming	Enables real-time monitoring of soil conditions	[19]
Clay Nanocomposite	Improved ion-exchange capacity, stability	Salinity management, nutrient delivery	Reduces salt stress, enhances nutrient availability	[77,89,129]

**Table 14:** Environmental Benefits of Hydrogel Use.

Environmental Benefit	Mechanism of Action	Agricultural Application	Citation
Water Conservation	Improved soil moisture retention, reduced irrigation frequency	Drought-prone regions, arid climates	[9,100,116,120]
Soil Erosion Control	Stabilized soil structure, reduced surface runoff	Sloped or degraded lands	[16,69]
Reduced Fertilizer Runoff	Controlled nutrient release, improved nutrient efficiency	Intensive farming systems	[6,22,80,89]
Enhanced Soil Health	Better soil aeration, improved microbial activity	Organic farming, sustainable agriculture	[24,36]

## Data Availability

Data sharing is not applicable to this article as no new data were created or analyzed in this study.
